# Tescalcin promotes highly invasive papillary thyroid microcarcinoma by regulating FOS/ERK signaling pathway

**DOI:** 10.1186/s12885-022-09643-9

**Published:** 2022-05-31

**Authors:** Xiuhe Zou, Qian Zhou, Yan Nie, Junhe Gou, Jing Yang, Jingqiang Zhu, Zhihui Li, Yanping Gong

**Affiliations:** 1 Thyroid and Parathyroid Surgery Center, West China Hospital, Sichuan University, No. 37 Guo Xue Xiang, Chengdu, 610041 Sichuan China; 2 West China School of Medicine, Sichuan University, Chengdu, China; 3 Department of pathology, West China Hospital, Sichuan University, Chengdu, China

**Keywords:** Tescalcin, Papillary thyroid microcarcinoma, Invasion, Metastasis, FOS, ERK1/2

## Abstract

**Background:**

Part of papillary thyroid microcarcinoma (PTMC) has a high risk of tumor invasion and metastasis, which may occur in the regional lymph node metastasis or distant metastasis, severely threatening the life of patients. Invasion and metastasis are tightly involved in the proliferation, migration and invasion in cancer. This study aimed to investigate the role of tescalcin (TESC) in the proliferation, migration and invasion of PTMC.

**Methods:**

The expressions of TESC in PTMC tissues and cells were detected by immunohistochemistry or qRT-PCR. Then, TPC-1 and BHT101 cells transfected with TESC-RNAi were used for the transcriptome sequencing. The proliferation, apoptosis, migration and invasion of TPC-1 and BHT101 cells were detected by CCK-8, colony formation, flow cytometric assay, transwell migration and scratch test. Moreover, TESC-RNAi transfected TPC-1 and BHT101 cells were subcutaneously injected into mice. Tumor volume and weight were calculated, and the positive rate of Ki-67 was determined by immunohistochemistry. Finally, the levels of c-Fos, ERK1/2 and p-ERK1/2 were determined by western blot.

**Results:**

The expressions of TESC in PTMC tissues and cell lines were prominently enhanced. Transcriptome sequencing results showed that c-Fos was decreased in TPC-1 and BHT101 cells transfected with TESC-RNAi, which was associated with multiple different signaling pathways including the MAPK signaling pathway. Furthermore, TESC promoted the progress of PTMC by regulating the expression of c-Fos, which might be associated with the ERK signaling pathway.

**Conclusions:**

TESC promoted the growth and metastasis of PTMC through regulating c-Fos/ERK1/2.

## Background

In the last few decades, the occurrence of papillary thyroid carcinoma (PTC) has been increasing all over the world, with the incidence continually increasing between 1973 and 1977 and 1998–2002 [1, 2]. With the improved understanding of thyroid nodule disease and the advancement of ultrasound examination technology, the detectable rate of papillary thyroid microcarcinoma (PTMC) has increased in recent years [3]. According to World Health Organization (WHO), an incidental PTC with the longest diameter ≤ 1 cm was defined as PTMC [4]. Generally, patients diagnosed with PTMC have a favorable prognosis, and the risk of distant metastasis is relatively low, resulting in a mortality rate as low as 0.5% for PTMC [5]. Although most PTMC was low risk, it is unavoidable that a few PTMC possessed a high risk for tumor invasion and metastasis. Therefore, studying the aggressive PTMC was indispensable for offering the most comprehensive treatment.

Tescalcin (TESC), also known as calcineurin B homologous protein 3 (CHP3), was first discovered as a differentially expressed gene in the development of embryonic mouse testis [6]. Since TESC contained an EF-hand motif that is characteristic of a large family of Ca^2+^-binding proteins, it is supposed to be involved in cell growth and differentiation [7]. Previous studies have proved that abnormal expression of the TESC was associated with many types of malignant tumors including melanoma, gastric cancer, renal cell carcinoma, and colorectal cancer [8–10]. Importantly, researchers have demonstrated that altered the expression of TESC is also related to radiation-induced PTC in pediatric patients exposed to radioactive fallout from the Chernobyl disaster [11]. However, the role of TESC in the occurrence and development of PTMC remained unclear.

c-Fos, one of activator protein 1 (AP-1) component members, was involved in a variety of biological processes, such as cell cycle progression, proliferation, differentiation, migration, angiogenesis and apoptosis [12, 13]. Furthermore, c-Fos can act as a differentiation transcription factor for bone-resorbing cells and osteoclasts [14]. On the other hand, it also plays a momentous role in tumor formation and inhibition [15]. Knockdown of c-Fos was demonstrated to cause the increase of apoptosis and the alteration of apoptosis-associated proteins expression in MCF-7/ADR cells [16]. It was also reported that the colon cancer cells invasion was regulated via ERK/c-Fos/MMP-7 signaling axis, and the metastasis of colon cancer cells may be treated by the suppression of the ERK/c-Fos/MMP-7 signaling pathway [17].

In the present study, TESC was notably increased both in PTMC tissues and cells. Then, we conducted a series of experiments to investigate the regulative role of TESC mainly in TPC-1 and BHT101. Furthermore, the underlying molecular mechanism of TESC in PTMC has also been clarified.

## Methods

### Cells and tissue samples

Human thyroid carcinoma cell lines TPC-1, BHT101, 8305C and CAL-62, and human normal thyroid cell lines Nthy-ori 3–1 were purchased from Procell (Wuhan, China). Cells were cultured in DMEM (Sigma Aldrich, America) supplied with 0.1% FBS (HyClone; Cytiva) and 1% streptomycin and penicillin (Sigma Aldrich, America) and maintained at 37 °C with 5% carbon dioxide (CO_2_). A collection of 10 pairs of human PTMC tissues and corresponding PTMC-adjacent tissues were acquired from West China Hospital of Sichuan University according to the institutional guidelines. No patients had received chemotherapy or radiotherapy prior to surgical resection. Written informed consent was obtained for use of the tissues. This study was approved by the Ethics Review Board of West China Hospital of Sichuan University.

### Animals experiment

Four-week-old nude mice were purchased from Chengdu Dossy Experimental Animals CO., LTD. Mice were housed under pathogen-free conditions and in a temperature-controlled room illuminated for 12 h daily. Following acclimation for 1 week, mice were divided into the sh-NC and the sh-TESC group (*n* = 6). Mice in the sh-TESC group were subcutaneously injected TESC-RNAi transfected cells (a total of 1 × 10^6^), while mice in the sh-NC were subcutaneously injected with the same volume of negative control (NC)-RNAi transfected cells to build the xenograft model. Tumor formation was monitored by detecting tumor volumes and weights. The study was carried out in compliance with the ARRIVE guidelines. The protocol was approved by the Institutional Animal Care and Use Committee of Sichuan University. Animals received humane care in accordance with study guidelines established by the second affiliated hospital of Sichuan University.

### Immunohistochemical staining (IHC)

The tissues of PTMC and non-PTMC from patients were fixed in 4% paraformaldehyde at room temperature, embedded in paraffin wax, and cut into 5 μm sections. The sections were then dewaxed with xylene and ethanol gradient and had their endogenous peroxidase activities quenched using 3% hydrogen peroxide at room temperature for 10 min. Anti-TESC antibody (1:200, cat. no. 11125–1-AP, Proteintech, USA) was incubated with the sections overnight at 4 °C before the sections were incubated with biotinylated secondary antibodies at 37 °C for 30 min. Diaminobenzidine was used for histochemical reactions and hematoxylin was used for counterstaining. Histological examination was performed on a light microscope (Olympus, Japan) and the images were analyzed using the Image-Pro Plus software (Media Cybernetics, Inc., Rockville, MD, USA). In addition, tumor tissues of mice were collected and fixed with 4% paraformaldehyde. Then, paraffin sections (5 μm) were analyzed by IHC with anti-Ki-67 antibody (1:200, cat. no. ab16667, abcam, USA).

### Quantitative reverse transcriptase-polymerase chain reaction (qRT-PCR)

Total RNA was extracted from cell samples using TRIzol reagent (TaKaRa Biotechnology Co., Ltd., Dalian, China) according to the manufacturer’s specifications. The cDNA was synthesized with a PrimeScript RT reagent Kit (Takara, RR047A) with the manufacturer’s instruction. qRT-PCR was carried out by the Bio-Rad ScripTM cDNA Synthesis Kit (Bio-Rad Laboratories, Inc., Hercules, CA, USA). The primer sequences were shown as follows: TESC (Forward primer: 5′-GTCGGGAAACCCTCACATCG-3′, Reverse primer: 5′-CGAAGGTGATCCCCTCGTAC-3′), c-FOS (Forward primer: 5′-CCATTACAGCTGTGGCTACGAGT-3′, Reverse primer: 5′-GGTCTGTTCGATGTTCCTCAAG-3′), and GAPDH (Forward primer: 5′-TATCGGACGCCTGGTTAC-3′, Reverse primer: 5′-CGTTCAAGTTGCCGTGTC-3′). The qRT-PCR amplification conditions were as follows: 95 °C for 5 min, 95 °C for 15 s and 60 °C for 30 s of 40 cycles. GAPDH was the internal control. The level of genes was analyzed by the comparative threshold cycle method (2^-△△CT^ method), where ΔΔCT = ΔCT _treatment_- ΔCT _control_ and ΔCT = Ct _target_ - Ct _reference_.

### Cell transfection

Three target RNAi sequences of TESC (TESC-RNAi 1: GGCCTGGCTGATGAGATCAAT; TESC-RNAi 2: CCGGAAGGAGAAGCTGAGATT; TESC-RNAi 3: CCGCATCACTCTGGAAGAATA) and the negative control sequence (NC: TTCTCCGAACGTGTCACGT) were cloned into the GV493 vector (Genechem, China). Subsequently, the constructed vector was transfected into TPC-1 and BHT101 cells using Polybrene (Sigma Aldrich, America), respectively. The relative expression level of TESC was determined using qRT-PCR after 16 h. The most significant reduction in TESC expression was selected from the three TESC-RNAi for subsequent experiments.

The sequences of TESC were synthesized and inserted into vector plasmids pcDNA obtained from Vigene Biosciences (Rockville, America), and then was transfected into TPC-1 and BHT101 cells using Lipofectamine 3000 (Invitrogen, America) for following assays. The RNAi sequences of c-Fos (sense sequence: 5′-CACCGCTTCATTCCCACGGTCACTTTCAAGAGAAGTGACCGTGGGAATGAAGTTTTTTG-3′ and the antisense sequence: 5′-GATCCAAAAAACTTCATTCCCACGGTCACTTCTCTTGAAAGTGACCGTGGGAATGAAGC-3′) were cloned into GV493 vector (Genechem, China), and then transfected into TPC-1 and BHT101 cells using Polybrene (Sigma Aldrich, America), respectively.

### Cell counting Kit-8 assay

TPC-1 and BHT101 cells were inoculated in 96-well plates with a density of 1 × 10^5^/well and then maintained for 24 h at 37 °C in 5% CO_2_. Subsequently, the proliferation of cells was detected using the Cell Count Kit-8 (Dojindo Laboratories, Kumamoto, Japan) according to the manufacturer’s protocol. The absorbance was recorded at 450 nm by a microplate reader (ThermoFisher, America).

### Colony formation assay

TPC-1 and BHT101 cells were inoculated in 6-well plates with a density of 1 × 10^4^/well at 37 °C for 14 days. Then, the clone numbers were counted manually after colonies were immobilized and stained with 4% paraformaldehyde and 0.1% crystal violet (Sigma Aldrich, America) for 30 min, respectively.

### Cell transwell invasion and migration assay

Cells were digested and resuspended with 1 × 10^6^/ml after 24 h transfection. For the cell invasion, the transwell chamber was spread with the Matrigel matrix with serum-free medium dilution, and then appended with the cell suspension. Cells were hatched at 37 °C and 5% CO_2_. Cells were immobilized with 4% paraformaldehyde and stained with 0.1% crystal violet (Sigma Aldrich, America) for 15 min after 24 h culture. The images were photographed and counted using microscopy (Olympus, Japan). The scratch assay was used to detect cell migration. Cell suspension was seeded onto the 6-well plate after drawing horizontal lines on the back of the plate. And the sterile spearhead was used to scratch the cells vertically relative to the lines on the back of the plate. Cells were washed with PBS, and then cultured in fresh FBS-free medium for 24 h and cell migration was observed under a microscope (Olympus, Japan).

### Flow cytometry assay

TPC-1 and BHT101 cells after transfection were collected and stained with Annexin V-APC and PI (Sigma Aldrich, America) at room temperature for 20 min in the dark. The fluorescence of the cells was measured by flow cytometry (BD FACSVerse, America).

### RNA-seq library preparation and Illumina sequencing

The total RNA from TPC-1 and BHT101 cells samples treated with TESC-RNAi or NC-RNAi was extracted using TRIzol reagent (TaKaRa Biotechnology Co., Ltd., Dalian, China) according to the manufacturer’s specifications. The integrity and purity of the acquired RNA were analyzed by an Agilent BioAnalyzer 2100. The mRNA-Seq libraries were established using an Illumina TruSeq RNA Sample Prep Kit (Beijing Novogene Zhiyuan Technology Co., Ltd., China). Briefly, the mRNA was purified from total RNA by poly-T oligo-attached magnetic beads, and then the purified mRNA was reversely transcribed into cDNA. The Illumina HiSeq 2500 sequencing platform was employed to perform the sequencing in paired-end reads after cDNA was linked to the adaptor and amplified using PCR. The reads were mapped to the *Homo sapiens* genome using TopHat v1.4.1 with default parameters (*−r 400 -p 8*) after mRNA-Seq data were preprocessed. The quality of data from mRNA sequencing was evaluated using the FastQC method (http://www.bioinformatics.babraham.ac.uk/projects/fastqc/).

### Bioinformatics analysis of mRNA-seq data

Trimmomatic (v0.30) was utilized to remove the adaptor sequences and the low-quality (< 20) bases at the 5′ and 3′ ends. Subsequently in order to obtain the clean reads ≥70 bp for subsequent analysis. The RSEM (https://www.biostat.wisc.edu/~cdewey/) was employed to analyze the gene abundances. The differentially expressed genes (DEGs) between two groups were obtained after each gene expression was quantified according to FPKM values (expected number of fragments per kilobase of transcript sequence per million base pairs sequenced) mapped to the clean reads. The DGEs from TPC-1 and BHT101 cells were screened, and then intersections were taken (control group and gene interference group|log2 fold change (FC)| ≥ 1.5). The screened differential expression bases are verified in TGCA. The DEGs in GO functional enrichment and Kyoto Encyclopedia of Genes and Genomes (KEGG) pathway [18] were analyzed using Ami GO with the default parameters and Cytoscape with the ClueGO plugin. The obtained DEGs were performed the gene enrichment analysis using DAVID or metascape software.

### Western blot analysis

Cells were collected and lysed using RIPA lysis buffer (Boster, Wuhan, China) containing 1% protease inhibitors, and the concentrations of proteins were calculated using the BCA protein quantification kit (Abcam, UK) in accordance with the manufacturer’s specifications. Then, protein samples were separated with 12% SDS-PAGE and electrically transferred onto PVDF membranes (EMD Millipore, Billerica, MA, USA). The membranes were blocked in TBST (Sigma Aldrich, America) (containing 5% skimmed milk) at room temperature for 1 h, and then incubated with primary antibodies at 4 °C overnight. After rinsing with TBST for 3 × 5 min, the membrane was incubated with goat-anti-rabbit IgG (H + L)-HRP (1: 5000, 31,460, Thermo Fisher, diluted in TBST containing 5% skimmed milk) for 1 h at room temperature. The reaction was visualized using an enhanced chemiluminescence detection kit DAB (Bio-Rad Laboratories, Inc.). The gray value was obtained using Image-ProPlus software (Media Cybernetics, Inc., Rockville, MD, USA). The primary antibodies used were as follows: c-Fos (cat. no. 4384), ERK1/2 (cat. no. 9102), p-ERK1/2 (cat. no. 9101), E-cadherin (cat. no. 3195), N-cadherin (cat. no. 13116), Vimentin (cat. no. 5741) and Snail (cat. no. 3879), and β-actin (cat. no. 4970; Cell Signaling Technology, Inc., Danvers, MA, USA) at 1: 1000 dilutions.

### Statistical analysis

Data were shown as the means ± SD. The student’s t-test was used to analyze the data with only two groups, while the one-way analysis of variance was used to analyze the differences among multiple groups by the SPSS 22.0 statistical software (IBM, Armonk, New York, USA) followed by *Post-hoc* Bonferroni test. The differences were considered statistically significant when *p* < 0.05.

## Results

### TESC facilitated proliferation***,*** migration, and invasion of PTMC

**Fig. 1:**
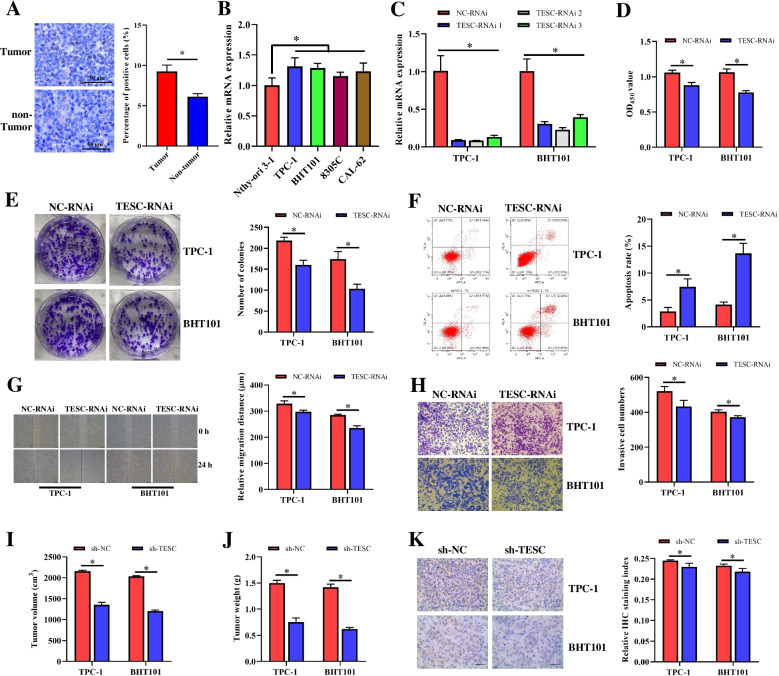
TESC effectively promoted the proliferation, migration, invasion, and inhibited the apoptosis of PTMC. **A** The expression of TESC in PTMC tissues and PTMC-adjacent tissues was detected by IHC, and the expression change of TESC was statistically analyzed. **B** The mRNA expression of TESC in the cells was detected using qRT-PCR. **C** Three target RNAi sequences of TESC were cloned into GV493 vector, and then were transfected into TPC-1 and BHT101 cells using Polybrene, respectively. The interference efficiency was determined by qRT-PCR. **D** and **E** The effect of TESC on the proliferation of TPC-1 and BHT101 cells was detected by CCK8 and colony formation assay. **F** The apoptosis rate of TPC-1 and BHT101 cells was determined by flow cytometric assay. **G** The effect of TESC on the migration of TPC-1 and BHT101 cells was examined by scratch test. **H** The effect of TESC on the invasion abilities of TPC-1 and BHT101 cells was examined by transwell assay. **I** and **J** TESC-RNAi transfected (a total of 2 × 10^6^) or control cells were subcutaneously injected into the flanks of nude mice. Tumor volume and weight were measured. **K** The level of Ki-67 was detected by IHC after the inhibition of TESC. The means ± SD of three independent samples were shown. *p < 0.05

To reveal the expression pattern of TESC in PTMC, the human PTMC tissues and corresponding PTMC-adjacent tissues were subjected to detect the TESC expression by IHC. As shown in Fig. 1A, the percentage of positive cells in human PTMC tissues was significantly increased compared with the corresponding PTMC-adjacent tissues, suggesting that TESC protein was significantly up-regulated in human PTMC tissues. And the mRNA expressions of TESC in the human thyroid carcinoma cell lines TPC-1, BHT101, 8305C and CAL-62 were also prominently enhanced compared to that in the human normal thyroid cell line Nthy-ori 3–1 (Fig. 1B). Moreover, to investigate the role of TESC in PTMC, TPC-1 and BHT101 were selected for subsequent studies, and the expression of TESC was interfered by three TESC-RNAi. The interference efficiency was assessed using qRT-PCR. The results showed that all three TESC-RNAi can effectively inhibit the level of TESC, of which the interference efficiency of TESC-RNAi 2 was the best both in TPC-1 and BHT101 cells (Fig. 1C). Thus, TESC-RNAi 2 was used for further assays and designated as TESC-RNAi. Then, CCK-8 and colony formation assays revealed that TESC-RNAi observably declined the proliferation of TPC-1 and BHT101 (Fig. 1D and E). The apoptosis rate of TPC-1 and BHT101 cells was dramatically promoted by TESC-RNAi according to flow cytometry assay (Fig. 1F). And the migration and invasion abilities of TPC-1 and BHT101 cells were markedly inhibited by TESC-RNAi (Fig. 1G and H). Moreover, after mice were subcutaneously injected with TESC-RNAi transfected TPC-1 and BHT101 cells, the tumor volume and weight were significantly reduced, and the positive rate of Ki-67 was also signally lower than of the sh-NC group (Fig. 1 I-K). Briefly, these results suggested that TESC effectively promoted the proliferation, migration and invasion, and suppressed the apoptosis of TPC-1 and BHT101 cells.

### C-Fos was down-regulated in TPC-1 and BHT101 cells transfected with TESC-RNAi

**Fig. 2:**
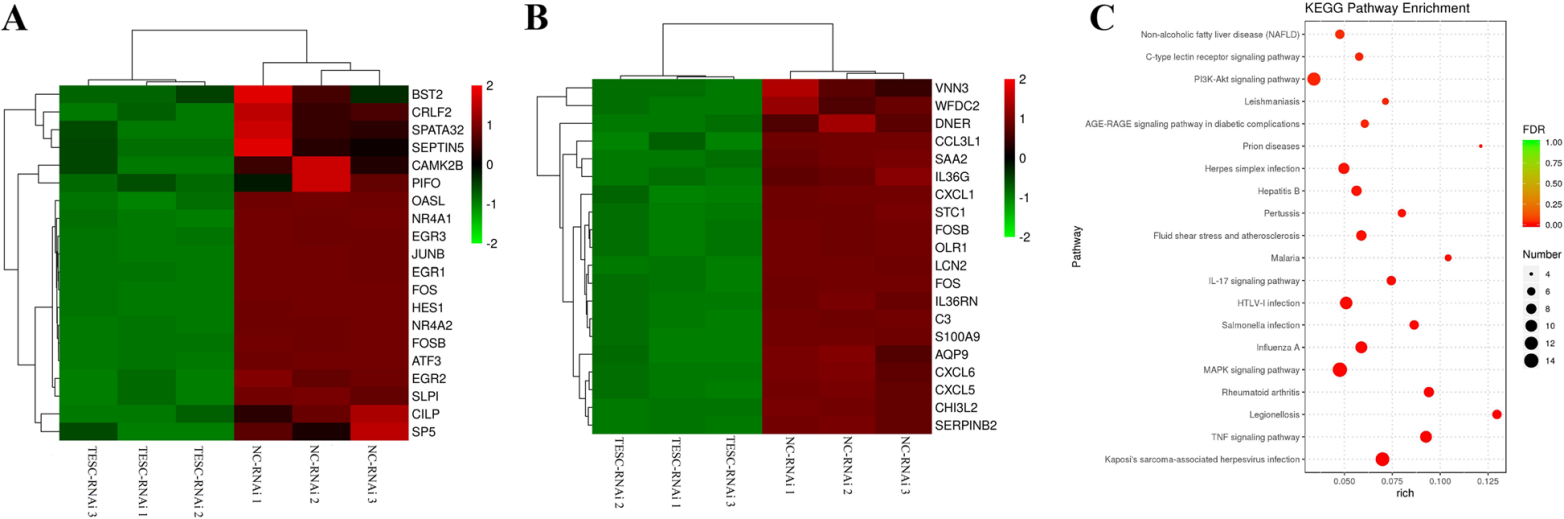
GO and KEGG enrichment analysis of TPC-1 and BHT101 cells transfected with TESC-RNAi. The target RNAi sequences of TESC was cloned into GV493 vector, and then were transfected into TPC-1 and BHT101 cells using Polybrene, respectively. Subsequently, TPC-1 and BHT101 cells were executed the transcriptome sequencing. **A** and **B** GO enrichment analysis of DEGs in TPC-1 and BHT101 cells, respectively. **C** KEGG enrichment analysis of an intersection of TPC-1 and BHT101 cells

After TESC-RNAi were transfected into TPC-1 and BHT101 cell lines to knockdown TESC respectively, transcriptome sequencing was executed for these two cell lines. c-Fos was found to be down-regulated both in TPC-1 and BHT101 cells according to TOP 20 of down-regulated genes (Fig. 2 A and B). Thus, we further analyzed the top 10 enriched KEGG pathways of c-Fos both in TPC-1 and BHT101 cells. According to the c-Fos enrichment pathways, the intersection of the two cell lines was taken to determine a KEGG diagram. As shown in Fig. 2C, the enriched KEGG pathways involved in c-Fos mainly contained MAPK signaling pathway, PI3K-Akt signaling pathway, TNF signaling pathway, non-alcoholic fatty liver disease, C-type lectin receptor signaling pathway and IL-17 signaling pathway. Thus, c-Fos was decreased in TPC-1 and BHT101 cells transfected with TESC-RNAi, which was associated with multiple different signaling pathways.

### A directly positive correlation between TESC and c-Fos in TPC-1 and BHT101 cells

**Fig. 3:**
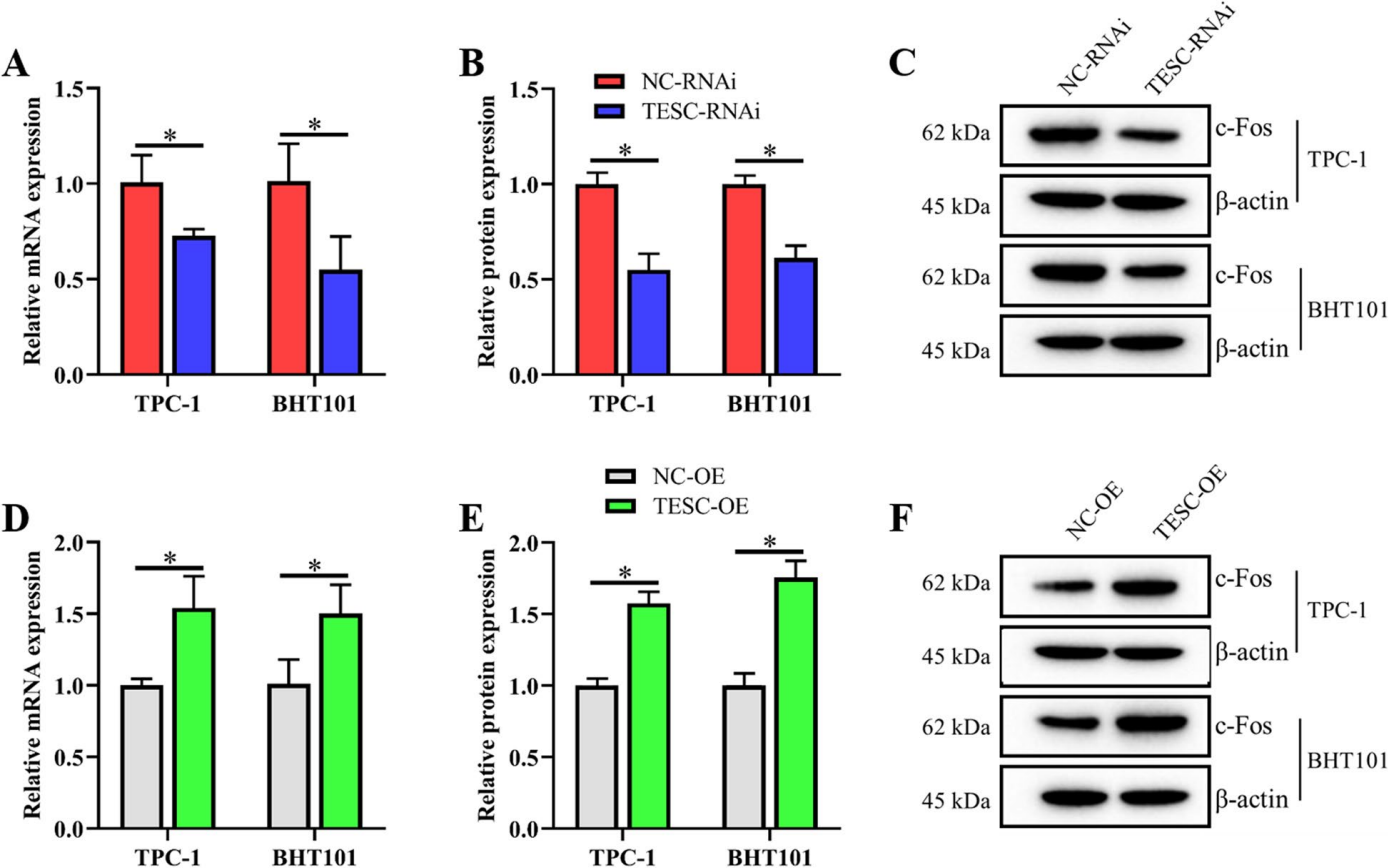
A directly positive correlation between TESC and c-Fos in TPC-1 and BHT101 cells. The mRNA and protein level of c-Fos were determined after the expression of TESC was downregulated and upregulated in TPC-1 and BHT101 cells respectively. **A** The relative mRNA level of c-Fos was detected using qRT-PCR after the inhibition of TESC in TPC-1 and BHT101 cells. **B** The relative protein level of c-Fos was shown as a bar graph after the inhibition of TESC in TPC-1 and BHT101 cells. **C** The proteins expression of c-Fos was examined by western blot assay after the inhibition of TESC in TPC-1 and BHT101 cells. **D** The relative mRNA level of c-Fos was detected using qRT-PCR after the overexpression of TESC in TPC-1 and BHT101 cells. **E** The relative protein level of c-Fos was shown as a bar graph after the overexpression of TESC in TPC-1 and BHT101 cells. **F** The proteins expression of c-Fos was examined by western blot assay after the overexpression of TESC in TPC-1 and BHT101 cells. Data in qRT-PCR assay were expressed after being normalized to GAPDH. Data in western blot assay were expressed after being normalized to β-actin. The means ± SD of three independent samples were shown. *p < 0.05

To verify the correlation between TESC and c-Fos, the mRNA and protein levels of c-Fos were determined after the expression of TESC was downregulated and upregulated in TPC-1 and BHT101 cells respectively. The downregulated and upregulated mRNA and protein expressions of c-Fos were accord with the inhibition and overexpression of TESC both in TPC-1 and BHT101 cells respectively, which indicated a positive correlation between TESC and c-Fos in TPC-1 and BHT101 cells (Fig. 3).

### TESC promoted the progress of PTMC by regulating the expression of c-Fos

**Fig. 4:**
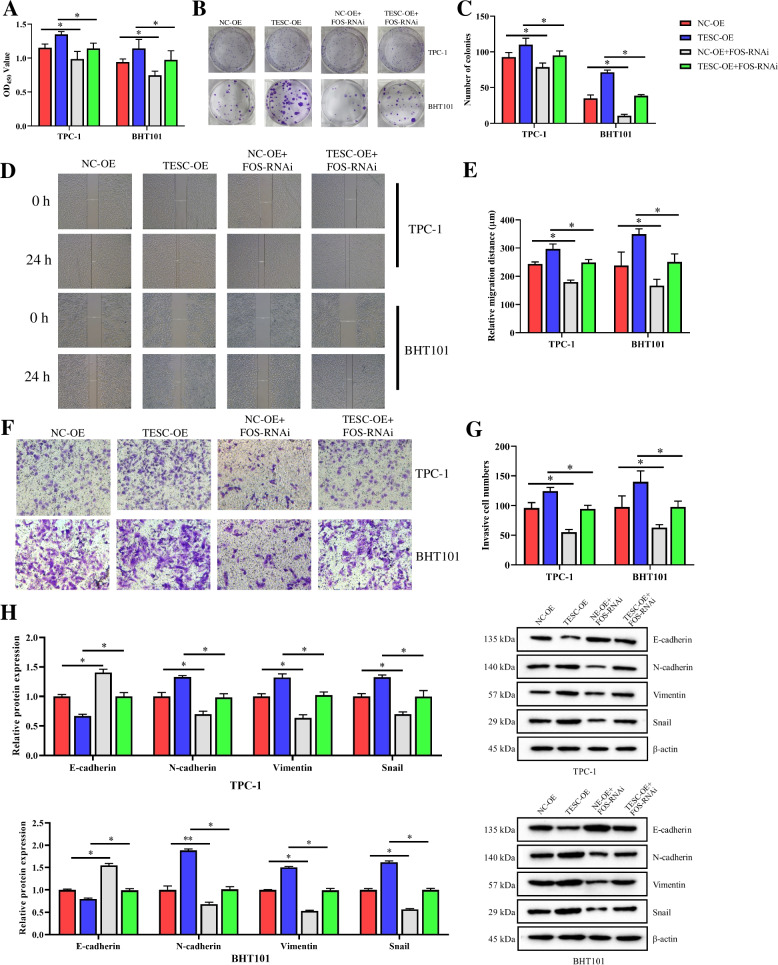
TESC promoted the progress of PTMC by regulating the expression of c-Fos in TPC-1 and BHT101 cells. **A**-**C** The effect of TESC on the proliferation of TPC-1 and BHT101 cells was detected by CCK8 and colony formation assay. **D** and **E** The effect of TESC on the migration of TPC-1 and BHT101 cells was examined by scratch test. **F** and **G** The effect of TESC on the invasion abilities of TPC-1 and BHT101 cells was examined by transwell assay. **H** The proteins expressions of EMT associated proteins E-cadherin, N-cadherin, Vimentin and Snail were examined by western blot assay. Data in western blot assay were expressed after being normalized to β-actin. The means ± SD of three independent samples were shown. *p < 0.05

Then, whether the progress of PTMC was significantly regulated after TESC targeting to c-Fos was investigated. As shown in Fig. 4, the proliferation, migration and invasion abilities of TPC-1 and BHT101 were significantly increased after overexpression of TESC, which was reversed by FOS-RNAi. Meanwhile, the proliferation, migration and invasion abilities of TPC-1 and BHT101 in the NC-OE + FOS-RNAi group were prominently declined compared to these in NC-OE group, suggesting the effectiveness of FOS-RNAi in inhibiting the proliferation, migration and invasion of TPC-1 and BHT101 (Fig. 4). Collectively, these results indicated TESC promoted the progress of PTMC by regulating the expression of c-Fos.

### Interaction of TESC and c-Fos via ERK1/2

**Fig. 5:**
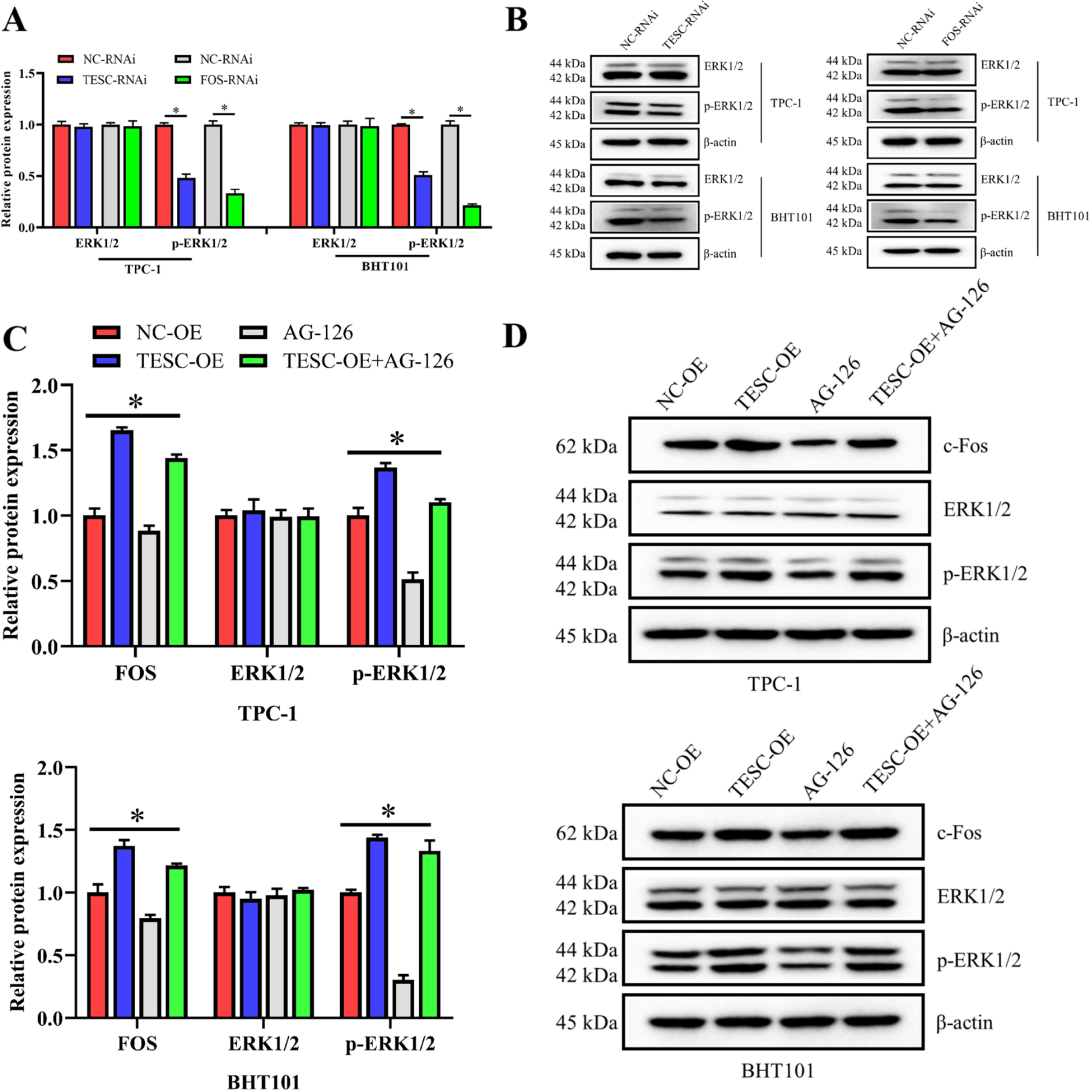
TESC interacted with c-Fos through ERK1/2 in TPC-1 and BHT101 cells. **A** The relative protein level of ERK1/2 and phosphorylated ERK1/2 was examined by western blot assay in TPC-1 and BHT101 cells treated with TESC-RNAi or FOS-RNAi. **B** The relative protein level of ERK1/2 and phosphorylated ERK1/2 was shown as a bar graph in TPC-1 and BHT101 cells treated with TESC-RNAi or FOS-RNAi. **C** Overexpressed plasmid of TESC was transfected into TPC-1 and BHT101 cells, and then cells were treated with ERK inhibitor AG-126. The proteins expression of c-Fos, ERK1/2 and phosphorylated ERK1/2 was examined by western blot assay. **D** The relative protein level of c-Fos, ERK1/2 and phosphorylated ERK1/2 was shown as a bar graph. Data in western blot assay were expressed after being normalized to β-actin. The means ± SD of three independent samples were shown. *p < 0.05

According to the transcriptome sequencing analysis, TESC interacted with c-Fos might associate with multiple different signaling pathways including the MAPK signaling pathway. Therefore, we detected the protein expression of ERK, one of MAPK, in TESC-depleted and in FOS-depleted cells respectively. The results showed that the expression of p-ERK1/2 in the TESC-RNAi and FOS-RNAi was significantly decreased compared with the NC-RNAi group, confirming that TESC and c-Fos may be closely related to the MAPK cascade (Fig. 5A and B). Then, ERK inhibitor AG-126 was used to investigate the role of ERK1/2 in the interaction of TESC and c-Fos. Consistently, overexpression of TESC notably enhanced the protein level of c-Fos both in TPC-1 and BHT101 cells. However, the use of ERK inhibitor AG-126 dramatically reduced the upregulated protein level of c-Fos caused by overexpression of TESC both in TPC-1 and BHT101 cells (Fig. 5C and D). Meanwhile, the protein level of c-Fos in the AG-126 group was markedly lower than these in the NC-OE group both in TPC-1 and BHT101 cells (Fig. 5C and D). In addition, the phosphorylated level of ERK1/2 was observably increased after overexpression of TESC, which was markedly reversed by AG-126 treatment (Fig. 5C and D). Taken together, TESC maybe interact with c-Fos through ERK1/2 in TPC-1 and BHT101 cells.

## Discussion

PTMC is the solid malignant tumor with the fastest annual growth rate [19]. The explosive growth has been made PTMC occupy an important position in the surgical treatment of thyroid cancer in many clinical centers. PTMC that possessed high risk for tumor invasion and metastasis has been received more and more attention. Invasion is tightly involved in the proliferation, migration and invasion in cancer. TESC, a Ca^2+^ sensor plays a crucial role in driving cell growth and differentiation programs [7]. Furthermore, the altered expression of TESC has been reported in radiation-induced PTC in pediatric patients [11]. In the present study, we found TESC could act as a potential biomarker of PTMC, and investigated the effect and molecular mechanism of TESC on PTMC proliferation, migration and invasion.

TESC was found to be upregulated in both thyroid cancer tissues and cells in this study, which was the same as various cancers, including colorectal cancer, melanoma, renal cell carcinoma and gastric cancer [8, 10, 20]. Although several molecular markers of PTMC, including BRAF^V600E^ [21], TERT [22] and Thyroglobulin [23] have been used in the clinics, the practicality is still controversial due to diverse reasons. Therefore, TESC maybe an alternative prognostic biomarker in PTMC, in which a large number of studies would be required investment.

TESC was demonstrated to participate in the cell proliferation and differentiation of primary megakaryocytes, and suppression of TESC inhibited megakaryocytic differentiation [24]. Moreover, the effect of TESC on cancer development was investigated in a variety of tumors as well. For instance, downregulation of TESC declined the colorectal cancer cell migration and invasion through the suppression of the EMT and MMP signaling pathway [9]. The growth and metastasis of HT29 (colon cancer) and AGS (gastric cancer) cells were inhibited by TESC interference [25]. Repression of TESC suppressed the growth and metastasis of renal cell carcinoma through downregulating NHE1 and NF-kB signaling [10]. In accordance with these previous results, our data revealed that inhibition of TESC through small interference RNA prominently restrained the proliferation, migration and invasion of TPC-1 and BHT101 cells. Furthermore, the sensibility of Ki-67 was positively related to the proliferation of tumors [26]. The volume and weight of tumor, and the relative IHC staining index of Ki-67 ki-67 were significantly lower with TESC-RNAi treatment in vivo, which also confirmed that downregulation of TESC repressed cell growth. Thus, TESC promoted the proliferation and metastasis of TPC-1 and BHT101 cells.

TESC was shown to directly bind several partners and effectors, including Na^+^/H^+^ exchanger NHE1, protein kinase glycogen-synthase kinase 3 (GSK3) and subunit 4 of the COP9 signalosome (CSN4) [7]. In the present study, the expression of c-Fos was prominently reduced in both TESC-RNAi transfected TPC-1 and BHT101 cells through transcriptomic technology, which was confirmed in TPC-1 and BHT101 cells treated with knockdown or overexpression of TESC. Thus, there may be a positive interaction existing between TESC and c-Fos. The level of c-Fos was correlative with the clinicopathologic characteristics including sex, grade, perineural invasion and stages, as well as prognosis in pancreatic cancer [27]. Our results showed that the upregulated proliferation, migration and invasion abilities of TPC-1 and BHT101 caused by overexpression of TESC were significantly declined through the inhibition of c-Fos. Thus, TESC promoted the growth and metastasis of PTMC by regulating the expression of c-Fos in TPC-1 and BHT101 cells.

ERK1/2 located at the terminal kinase in the MAPK signaling pathway can be transported to the nucleus to modulate various biological functions, including growth, differentiation, migration and apoptosis [28]. Moreover, ERK1/2 is closely associated with the process of cancer. It has been reported that ERK1/2 plays an important role in tumor proliferation, invasion and metastasis, migration and angiogenesis [29]. In the present study, TESC-RNAi and FOS-RNAi significantly reduced the expression of p-ERK1/2 in TPC-1 and BHT101 cells. In addition, the protein level of c-Fos was notably enhanced via overexpression of TESC, which was reversed by the ERK inhibitor AG-126 treatment, thereby TESC targeting c-Fos through ERK1/2. Furthermore, pharmacological interference of ERK1/2 inhibited the TESC upregulation, which suggested that TESC acts downstream of ERK1/2 [24]. Thus, we speculated there may be a feedback ERK1/2/TESC/FOS/ERK1/2 signaling axis, which was needed further studies.

In summary, our results showed that the level of TESC was enhanced in thyroid cancer tissues and cells. In addition, TESC promoted the growth and metastasis of TPC-1 and BHT101 cells by regulating the expression of c-Fos. Meanwhile, there may be a TESC/FOS/ERK1/2 signaling axis in the TPC-1 and BHT101 cells. Previous studies have demonstrated that the expression of ERK was enhanced in a variety of cancer [30–32]. Blockade of the level of ERK signaling pathway repressed the growth of cancer cells [33]. Thus, the effect of ERK1/2 inhibitor on the growth and metastasis of PTMC might need further research. Briefly, the results provided a theoretical basis for the clinical development of diagnosis and therapy for PTMC.

## Data Availability

The datasets generated and/or analyzed during the current study are not publicly available due the data also forms part of an ongoing study but are available from the corresponding author on reasonable request.
